# New receptors for common MAMPs: Can wild relatives save citrus from disease?

**DOI:** 10.1093/plphys/kiad306

**Published:** 2023-05-23

**Authors:** Hannah M McMillan

**Affiliations:** Assistant Features Editor, Plant Physiology, American Society of Plant Biologists, USA; Department of Biology, Duke University, Durham, NC 27708, USA

As part of their response to microbes, plants recognize conserved microbial features, which triggers a wave of downstream signaling events (Ngou et al. 2022). These microbe-associated molecular patterns (MAMPs) include flagellin and chitin among other proteinaceous and structural elicitors (Ngou et al. 2022). Decades of research in model systems like Arabidopsis have revealed specific epitopes from these bacterial elicitors and their corresponding plant receptors as well as the downstream signaling events that lead to plant defense responses (Ngou et al. 2022). For example, the 22-amino acid epitope from flagellin, flg22, is recognized by the plant receptor FLS2, triggering a signaling cascade that contributes to defense responses such as a reactive oxygen species (ROS) burst, mitogen-activated protein kinase (MAPK) activation, callose deposition, and global transcriptional reprogramming (Gómez-Gómez and Boller 2000). These defense responses are designed to eliminate or contain pathogens and prevent damage to local and systemic plant tissue. Research in model systems has provided a critical framework for understanding plant immune responses and revealed many possible targets for breeding programs to improve plant resistance to infection. However, research is now needed to confirm known receptor homologs in crops and identify novel MAMP receptors that may not be present in model systems.

Indeed, newly emerging pathogens such as *Candidatus* Liberibacter asiaticus (*C*Las) have proven difficult to study despite our extensive knowledge of plant immunity in model systems (Ghosh et al. 2023). *C*Las is a phloem-colonizing bacterium transmitted by an insect vector, the Asian citrus psyllid, that causes citrus greening disease, or Huanglongbing (HLB) disease, in citrus (Ghosh et al. 2023). Citrus is the most extensively produced fruit crop worldwide, and Florida is the second largest producer of orange juice in the world (Hodges and Spreen 2012; USDA 2020). Without control measures, HLB results in an approximately $300 million economic loss (or 36.42% production value) per season to Florida's citrus industry (Monzó and Stansly 2020). If we expand this statistic to the approximately $3.4 billion yearly citrus production value in the United States, HLB results in roughly $1.2 billion in annual losses (USDA 2020). Research on HLB and *C*Las has progressed slowly for many reasons, including the long lifespan of perennial trees, the large amount of greenhouse or field space required to study the disease, reduced access to diverse genotypes among cultivated citrus varieties, a lack of genomic resources for citrus, and the added challenge that *C*Las cannot be cultured in vitro (Trinh et al. 2023). Studies have revealed that one possible microbial elicitor from *C*Las and other Liberibacter species is a 22-amino acid epitope of the bacterial cold shock protein (csp22), which in some plant species can be detected by the CORE receptor-like kinase receptor (Wang et al. 2016). Despite these advances, the rapid and extensive spread of HLB disease across the world and its resulting economic impact highlight a need to explore plant immune responses in non-model crop species in hopes of identifying new and effective control measures.

In this issue of *Plant Physiology*, Trinh et al. investigate 86 genotypes of the Rutaceae family, which includes wild and all cultivated varieties of citrus, to probe natural variation in the perception and magnitude of response to conserved microbial features (Trinh et al. 2023). These 86 genotypes capture a wide range of morphologic and phylogenetic diversity (Fig. 1A). Using assays for ROS generation and MAPK activation in response to flg22, chitin, or csp22 elicitors, their study reveals that 75/86 genotypes generate an immune response to chitin, 40 genotypes respond to flg22, and 45 respond to csp22 (Fig. 1B) (Trinh et al. 2023). Indeed, 24 of the tested genotypes respond to all 3 elicitors (Fig. 1B) (Trinh et al. 2023). Although these immune responses occur across many of the cultivated and wild citrus varieties, there is substantial variation in MAMP perception and the magnitude of MAMP responses even among closely related genotypes (Fig. 1B) (Trinh et al. 2023). Variation in perception and response also occurs in model systems including tomato and Arabidopsis (Veluchamy et al. 2014; Vetter et al. 2012) and could be the result of differences in receptor recognition of conserved features or downstream differences in signaling and transcription (Trinh et al. 2023).

**Figure 1.**
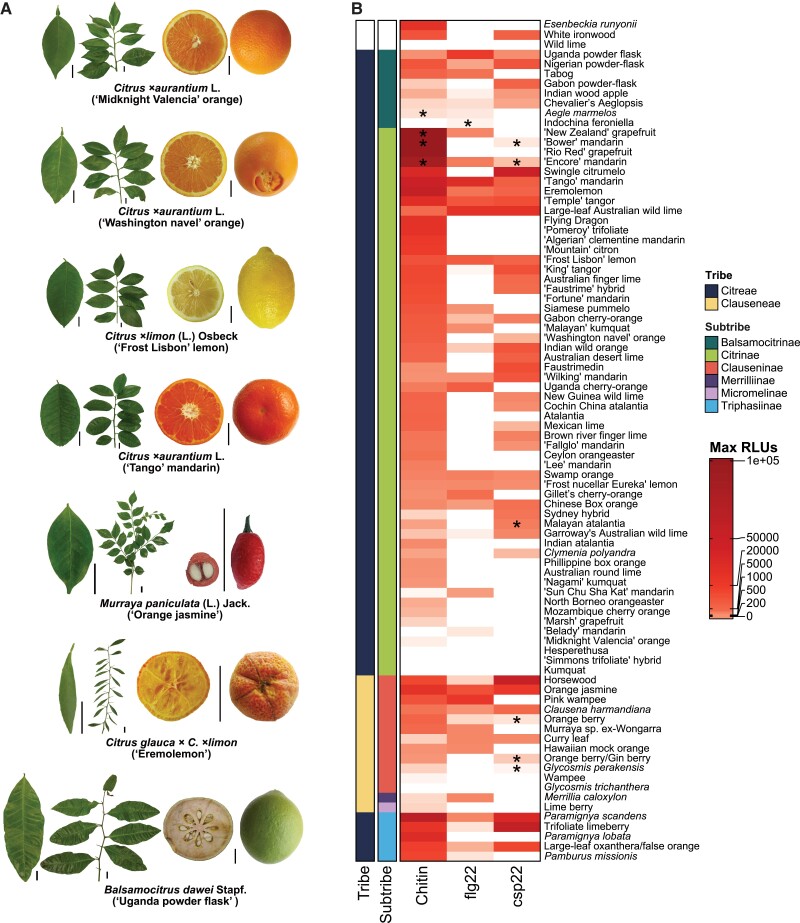
Genotypes within the Rutaceae family, including citrus, possess differing leaf morphologies and exhibit diverse responses to common MAMPs. **A)** Leaf, branch, and fruit morphologies of selected genotypes grown under greenhouse and field conditions. Scale bars = 2 cm. **B)** Heat map compiling average max relative light units (RLUs) from ROS assays in genotypes within the Rutaceae family, organized by MAMP and phylogenetic relationship. Max RLUs are averages of at least 3 independent experiments. Asterisks indicate genotypes that exhibit a variable response to the corresponding elicitor, where some but not all independent experiments responded with ROS. The MAMPs used are canonical features in the following concentrations: chitin (10 *μ*M), flg22 (100 nM), and csp22 (100 nM). Genotypes are referred to by common name unless otherwise unavailable. Figure panels and legend from Trinh et al. (2023).

To investigate further the differences in MAMP response, Trinh et al. identify homologs of known MAMP receptors from model organisms in responsive and non-responsive citrus genotypes and choose representative genotypes to explore in more detail (Trinh et al. 2023). The FLS2 receptor perceives flg22 and is conserved across many diverse plant families (Gómez-Gómez and Boller 2000; Ngou et al. 2022). In this study, *FLS2-1* and *FLS2-2* homologs were identified in “Washington navel” orange, a non-responsive genotype to flg22, and “Frost Lisbon” lemon, a responsive genotype (Trinh et al. 2023). In both genotypes, *FLS2-1* and *FLS2-2* showed induced expression in response to flg22 treatment, and transient expression assays in *Nicotiana benthamiana* showed that all *FLS2* homologs were able to elicit an ROS response to flg22 (Trinh et al. 2023). These results suggest that differences in flg22-mediated responses across the genotypes tested may not be regulated at the receptor level (Trinh et al. 2023). LYK5 and CERK1 receptors perceive chitin, but prior to this study their conservation across the plant kingdom was unknown (Ngou et al. 2022). Using an approach based on homology, phylogeny, and hidden Markov models, Trinh et al. found that 41% of citrus genotypes possess multiple *LYK5* homologs and 51% contain multiple *CERK1* homologs (Trinh et al. 2023). *LYK5* alleles from “Washington navel” orange and “Tango” mandarin encode conserved chitin binding residues and are expressed similarly in both genotypes, with and without chitin application (Trinh et al. 2023). Trinh et al. also functionally validated one *LYK5* allele that is identical in the two genotypes through complementation in the *lyk4/lyk5-2* Arabidopsis mutant, which restored the ROS and MAPK responses to chitin (Trinh et al. 2023).

Identifying possible receptors for csp22 in citrus posed a greater challenge. The tomato CORE receptor perceives csp22 and is able to generate resistance to *Pseudomonas syringae* pv tomato DC3000 when expressed in Arabidopsis (Wang et al. 2016). The closest CORE receptor homolog in citrus contained many polymorphisms, which could hinder recognition of the bacterial csp22 epitope (Trinh et al. 2023). Further, transiently expressed CORE receptor homologs from “Frost nucellar Eureka” lemon and “Washington navel” orange, both responsive genotypes to csp22, were unable to confer csp22 responsiveness in *N. benthamiana* (Trinh et al. 2023). This suggests that Rutaceae may possess an independently derived csp22 receptor (Trinh et al. 2023). Lack of response could occur due to mutations in the receptor but could also occur due to mutations in the bacterial epitope that allow pathogens to escape receptor recognition. The bacterial csp22 epitope from *C*Las contains several polymorphisms compared to the canonical csp22 sequence (Trinh et al. 2023). Intriguingly, while most of the Rutaceae genotypes that perceive csp22 do not respond to csp22 from *C*Las, members from the Balsamocitrinae and Clauseninae subtribes, including Uganda powder-flask and *Clausena harmandiana*, can perceive both csp22 epitopes (Trinh et al. 2023). This presents an exciting opportunity to test receptors from these broadly responsive genotypes in susceptible citrus cultivars for their ability to confer resistance and reduce HLB severity.

The results presented in this study show that MAMP perception and response varies greatly across even closely related citrus genotypes. The authors also demonstrate that some genotypes have enhanced responses to several distinct MAMP elicitors and polymorphic pathogen features. Given that heterologous expression of receptors can confer pathogen resistance in non-responsive genotypes, shown here and in other studies, these findings offer hope that new MAMP receptors can be identified and used in breeding programs to reduce disease severity. This is particularly promising in light of newly emerging pathogens, such as *C*Las, that are challenging to study by traditional methods. Boosting plant immune responses in this way could contribute to durable resistance that is not easily overcome by pathogen evolution.

## References

[kiad306-B1] Ghosh D , Kokane S, Savita BK, Kumar P, Sharma AK, Ozcan A, Kokane A, S SANTRA. Huanglongbing pandemic: current challenges and emerging management strategies. Plants. 2023:12(1): 160. 10.3390/plants12010160 PMC9824665 36616289

[kiad306-B2] Gómez-Gómez L , Boller T. FLS2: an LRR receptor–like kinase involved in the perception of the bacterial elicitor flagellin in Arabidopsis. Mol Cell. 2000:5(6):1003–1011. 10.1016/S1097-2765(00)80265-8 10911994

[kiad306-B3] Hodges AW , Spreen T. Economic impacts of citrus greening (HLB) in Florida, 2006/07-2010/11. Electronic Data Information Source (EDIS). 2012:FE903.

[kiad306-B4] Monzó C , Stansly PA. Economic value of conservation biological control for management of the Asian citrus psyllid, vector of citrus huanglongbing disease. Pest Manag Sci. 2020:76(5):1691–1698. 10.1002/ps.5691 31756775

[kiad306-B5] Ngou BPM , Ding P, Jones JDG. Thirty years of resistance: zig-zag through the plant immune system. Plant Cell. 2022:34(5):1447–1478. 10.1093/plcell/koac041 35167697 PMC9048904

[kiad306-B6] Trinh J , Li T, Franco JY, Toruño TY, Stevens DM, Thapa SP, Wong J, Pineda R, De Dios AE, Kahn TL, et al Variation in microbial feature perception in the Rutaceae family with immune receptor conservation in citrus. Plant Physiol. 2023:193(1):689–707. 10.1093/plphys/kiad263 37144828 PMC10686701

[kiad306-B7] USDA. National Agricultural Statistics Service 2020. Citrus Fruits: 2020. Summary. https://www.nass.usda.gov/Publications/Todays_Reports/reports/cfrt0820.pdf

[kiad306-B8] Veluchamy S , Hind SR, Dunham DM, Martin GB, Panthee DR. Natural variation for responsiveness to flg22, flgII-28, and csp22 and Pseudomonas syringae pv. Tomato in heirloom tomatoes. PLoS One. 2014:9(9):e106119. 10.1371/journal.pone.0106119 25180693 PMC4152135

[kiad306-B9] Vetter MM , Kronholm I, He F, Häweker H, Reymond M, Bergelson J, Robatzek S, De Meaux J. Flagellin perception varies quantitatively in Arabidopsis thaliana and its relatives. Mol Biol Evol. 2012:29(6):1655–1667. 10.1093/molbev/mss011 22319159

[kiad306-B10] Wang L , Albert M, Einig E, Fürst U, Krust D, Felix G. The pattern-recognition receptor CORE of Solanaceae detects bacterial cold-shock protein. Nat Plants. 2016:2(12):16185. 10.1038/nplants.2016.185 27892924

